# Reversible Functional Changes Evoked by Anodal Epidural Direct Current Electrical Stimulation of the Rat Auditory Cortex

**DOI:** 10.3389/fnins.2019.00356

**Published:** 2019-04-12

**Authors:** Ana Cecilia Colmenárez-Raga, Iván Díaz, Marianny Pernia, David Pérez-González, José M. Delgado-García, Juan Carro, Ignacio Plaza, Miguel A. Merchán

**Affiliations:** ^1^Instituto de Neurociencias de Castilla y León, University of Salamanca, Salamanca, Spain; ^2^Division of Neurosciences, Pablo de Olavide University, Seville, Spain

**Keywords:** auditory brain responses, quantitative immunocytochemistry, Iba1, GFAP, c-Fos, cortical descending control, otoprotection, tinnitus

## Abstract

Rat auditory cortex was subjected to 0.1 mA anodal direct current in seven 10-min sessions on alternate days. Based on the well-known auditory cortex control of olivocochlear regulation through corticofugal projections, auditory brainstem responses (ABRs) were recorded as an indirect test of the effectiveness and reversibility of the multisession protocol of epidural stimulation. Increases of 20–30 dB ABR auditory thresholds shown after epidural stimulation reverted back to control levels 10 min after a single session. However, increases in thresholds revert 4 days after multisession stimulation. Less changes in wave amplitudes and threshold shifts were shown in ABR recorded contralaterally to the electrically stimulated side of the brain. To assess tissue effects of epidural electric stimulation on the brain cortex, well characterized functional anatomical markers of glial cells (GFAP/astrocytes and Iba1/microglial cells) and neurons (c-Fos) were analyzed in alternate serial sections by quantitative immunocytochemistry. Restricted astroglial and microglial reactivity was observed within the cytoarchitectural limits of the auditory cortex. However, interstitial GFAP overstaining was also observed in the ventricular surface and around blood vessels, thus supporting a potential global electrolytic stimulation of the brain. These results correlate with extensive changes in the distribution of c-Fos immunoreactive neurons among layers along sensory cortices after multisession stimulation. Quantitative immunocytochemical analysis supported this idea by showing a significant increase in the number of positive neurons in supragranular layers and a decrease in layer 6 with no quantitative changes detected in layer 5. Our data indicate that epidural stimulation of the auditory cortex induces a reversible decrease in hearing sensitivity due to local, restricted epidural stimulation. A global plastic response of the sensory cortices, also reported here, may be related to electrolytic effects of electric currents.

## Introduction

Invasive techniques, such as epidural electric stimulation (EES), have been recently used to treat brain diseases (Nahas et al., 2010; Balossier et al., 2013; Levy et al., 2016; Williams et al., 2016). These techniques allow us to precisely stimulate the brain of a patient with low current intensities. Electric stimulation of the brain has been effective in neuro-otological diseases such as auditory hallucinations (Koops et al., 2015), tinnitus (Zeng et al., 2015), aphasia (Cherney, 2011, 2016; Cherney et al., 2012), and auditory agnosia (Bestelmeyer et al., 2018). Among these clinical applications, electrostimulation currently stands out for its effectiveness in treating a wide range of central hearing disorders. However, to date, analysis of the effects of epidural electrostimulation of the AC has not been performed in animal models.

In the classical experiments of Robert Galambos, electrical pulses on the floor of the IV ventricle of cats induced a decrease in cochlear compound action potentials (Galambos reflex), demonstrating the functional modulation of the olivocochlear system of cochlear responses to sound (Galambos, 1956). Furthermore, the AC is known to dynamically regulate the Galambos reflex through its direct or indirect descending projections to the superior olivary complex (Mulders and Robertson, 2000; Horvath et al., 2003). Hence, the brain cortex can adjust and improve cochlear outputs after sound stimulation in real time. In mustached bats, such corticofugal modulation was masterfully demonstrated by Xiao and Suga (2002), by CM recordings, through shifts in frequency tuning after electrical stimulation of the AC. After blocking the AC by TTX application, the corticofugal effect on cochlear hair cell activation was demonstrated in gerbils by DPOAE (Jager and Kossl, 2016). Similarly, after cooling or lidocaine blocking of the AC in chinchillas, plastic, top-down regulation of the AC was demonstrated by CAP and CM recordings (León et al., 2012). In addition, previous results from our laboratory have shown that restricted ablation of the rat AC significantly increases auditory thresholds (hearing suppression) analyzed by ABRs (Lamas et al., 2013).

Epidural electric stimulation of the posterolateral region of the superior temporal gyrus has long been shown to induce hearing suppression in patients (Penfield and Perot, 1963). In a more recent study, conducted during presurgical functional brain mapping of patients with refractory epilepsy, it was shown that the electrical stimulation of the AC led to a significant decrease in contralateral evoked autoacoustic emission amplitude, indicating an effective cortical control of peripheral auditory receptor through the olivocochlear efferent system in humans (Perrot et al., 2006).

Hearing suppression and partial or total tinnitus compensation were also shown after implanting electric epidural electrodes on the secondary AC (over the posterior superior temporal gyrus) (Friedland et al., 2007). Overall, these findings, both in humans and in animal models, clearly indicate a hierarchical, top-down cortical control over the olivocochlear efferent system in various species of mammals, including humans. In this study, we hypothesize that anodal electric activation of the AC will increase auditory thresholds (hearing suppression). Thus, the first aim of the present study is to assess the functional effects of EES on the AC by analyzing the resulting modifications of short-latency auditory evoked responses through the corticofugal pathway.

Glial cells are known to react to brain lesions, neuronal overactivation (Stence et al., 2001; Pekny and Nilsson, 2005; Pascual et al., 2012) or electric field stimulation (Pelletier et al., 2015). Thus, the second aim of the present study was to analyze potential lesions and/or glial activation after anodal direct current EES in the cortex using well-known glial immunocytochemical markers (GFAP and Iba1).

Computer-based models of CNS electric field stimulation has shown that passing axons are better candidates for hyperpolarization or depolarization in the cerebral cortex than neuronal cell bodies (McIntyre and Grill, 2004). *In vitro* extracellular recordings in rat visual cortex slices after selective inactivation of cell bodies or axons also indicates that, under electrical fields, neurites are more excitable than neuronal cell bodies (Nowak and Bullier, 1998). Therefore, given the large system of horizontal connections between sensory areas (Stehberg et al., 2014), EES may induce extensive functional changes in the brain cortex. In addition c-Fos immunocytochemistry has been used as an activity marker which was overexpressed after electrical stimulation of the brain (Boix-Trelis et al., 2009). Thus, the third goal of this paper was to analyze cortical effects of repeated anodal EES by Nissl staining and c-Fos quantitative immunocytochemistry.

## Materials and Methods

### Experimental Groups

This study was conducted in strict accordance with Spanish regulations (Royal Decree 53/2013 – Law 32/2007) and European Union guidelines (Directive 2010/63/EU) on the care and use of animals in biomedical research. All surgeries were performed under monitored anesthesia (respiratory rate, body temperature, and oxygen saturation), and all efforts were made to minimize suffering.

**FIGURE 1:**
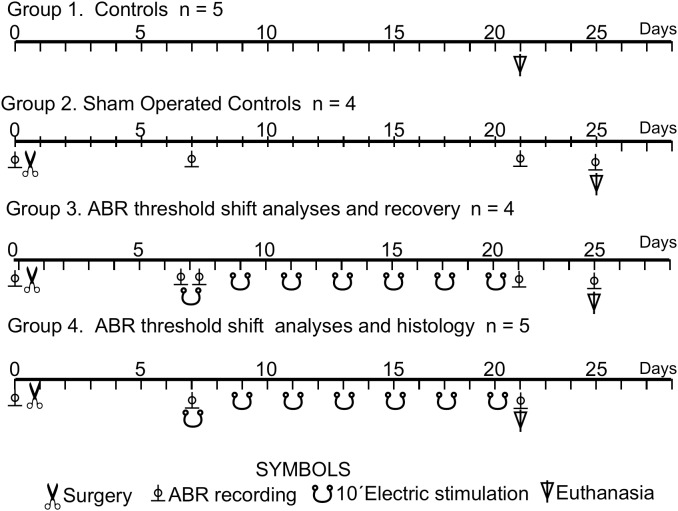
Experimental animal groups. Timeline of the multisession protocol of epidural stimulation of the AC. The meaning of the symbols is explained in the bottom of the figure.

In total, 18 young male Wistar rats weighing from 250 to 300 g were studied in four experimental groups: Group 1 controls (*n* = 5), Group 2 sham-operated controls (*n* = 4), Group 3 ABR threshold shift analyses and recovery (*n* = 4) and Group 4 ABR threshold shift and histological analysis (*n* = 5) (Figure 1). Eight cases with electrodes placed outside ACs coordinates were eliminated from the analysis.

### Surgery

**FIGURE 2:**
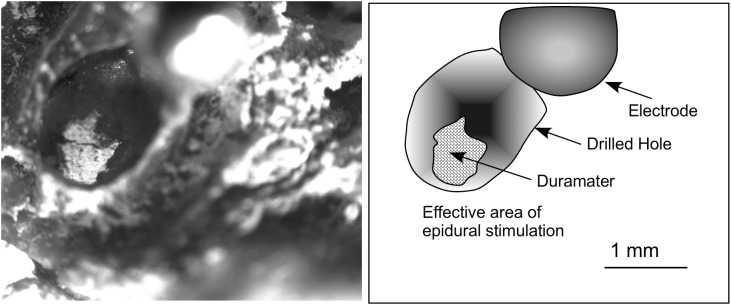
Superficial view of the skull after post-mortem electrode extraction (the electrode was twisted and is shown in the figure close to the drill bit on the right-hand corner, out of focus). Glued to a coverglass, the bone was placed in the microscope stage and transilluminated to take the picture. The contour of the surface of dura mater exposed to the electrode is clearly shown using this approach. Measures of the surface were used to correlate with EES effects on the brain surface and to estimate the amount of electric current injection.

Both sham-operated controls and stimulated rats were placed in a stereotaxic frame under gas anesthesia (2.5% isoflurane). The left temporal cranial surface was surgically exposed. Following the Paxinos and Watson atlas guide (Paxinos and Watson, 2005), four points were drawn on the surface of the bone delimiting the borders of the auditory area (we have previously published this surgical approach; for details, see Lamas et al., 2017). A round, 2-mm hole was drilled in the center of the square drawn on the bone surface until exposure of the surface of the dura mater (Figure 2). Cold saline (4°C) was dripping during the whole process to avoid thermal cortical lesions.

A silver ball electrode (anode) 1.2 mm in diameter was encrusted into the trepans, and two screws (cathode) were implanted in the contralateral side of the skull. After appropriately connecting the ball electrode and the screw using a connector, the device was fully covered by dental cement. Animals recovered from the anesthesia in their cages for 7 days before any further intervention (see time line protocol in Figure 1).

To calculate the surface of contact of the electrodes with the dura mater, after euthanasia, the skull was preserved, and the electrode carefully removed by drilling the dental cement of the pedestal. The bone was glued in parallel to a coverglass and transilluminated and photographed under a Leica DMRB microscope using a 2.5X long focal length objective. The effective contact surface was then measured from the microphotographs (Figure 2).

### Anodal Direct Current Stimulation

Using an ISU 200 BIP isolation unit controlled by a CS-20 stimulator (Cibertec, Madrid, Spain), a 0.1 mA constant anodal direct current was delivered for 10 min per session through the epidural bone-attached silver ball electrode. Stability of the voltage that was developed at the electrode due to the anodal current flowing through tissue resistance was monitored during the 10 min session using an oscilloscope. This current intensity was similar to that previously reported by some of us to produce a current intensity of 3.7 A/m^2^ in behaving rabbits (Márquez-Ruiz et al., 2012). The stimulation protocol was applied in awake animals of groups 3 and 4, seven times on alternating days (multisession EES) (Figure 1). For the extinction protocol (group 3), rats were similarly stimulated seven times but recorded right after the stimulation protocol and 4 days after the end of the last stimulation session. Controls and sham-operated rats (groups 1 and 2) were euthanized concomitantly with the stimulated animals of the EES groups.

### ABR Recordings

Recordings were performed under gas anesthesia using a real-time signal processing system (Tucker-Davis Technologies [TDT], System RZ-6, Alachua, Fl, United States). The system output was calibrated before the recordings using a one-quarter inch microphone (Brüel and Kjaer). Sound stimuli were 0.1 ms alternating polarity click, with a repetition rate of 21 bursts/s, delivered in 10 dB ascending steps from 10 to 90 dB, in an acoustically isolated chamber. The stimuli were delivered in close field using a magnetic speaker (TDT – MF1 Multi-Field Magnetic Speaker) connected to the ear through a 10 cm long plastic tube. This approach resulted in a total delay of 1.4 ms in stimulus arrival at the tympanic membrane. An ABR was obtained by averaging 1,000 EEG responses to 1,000 click stimuli. Evoked potentials were amplified and digitized using a Medusa RA16PA preamplifier and an RA4LI headstage. Three subcutaneous needle electrodes were placed at vertex and two mastoids. Monaural ABRs were recorded from vertex with reference to the mastoid ipsilateral to the click-stimulated ear. The needle at the mastoid contralateral to the stimulated ear served as ground electrode. Monaural ABRs were sequentially recorded with click stimulation to left and right ears. Placement of the recording electrodes was changed accordingly, i.e., in order to record the signals from the side of the sound stimulated ear. ABR recordings of both sides were analyzed separately. The final signal was filtered with a 500-Hz high-pass filter and a 3,000-Hz low-pass filter (for more details on the ABR recording methodology, see Lamas et al., 2013). The ABR threshold was defined as the minimal sound intensity that evoked a significant voltage change (in a latency range between 1.4 and 5 ms) exceeding the mean ± 2SD of the voltage value of background activity of the first ms of the recording. The absolute wave latency was defined as time in milliseconds from the stimulus onset to the positive peak of the wave. Wave II was firstly recorded in ABRs, and was used to calculate thresholds (©MatLab R 2017 A). The amplitudes of the ABR waveforms were measured as the peak-to-peak amplitude between the preceding negative trough to the subsequent positive peak of a given wave. Statistical analysis of thresholds was performed using the IBM^®^SPSS^®^software, version 20 (IBM Corp. and SPSS Inc., Chicago, IL, United States, **RRID:SCR_002865**). Paired *t*-test analysis of threshold values was performed, and differences were considered significant for *p*-values < 0.05.

### Histology

#### Fixation and Sectioning

After the stimulation protocol, the animal was deeply anesthetized with isoflurane and decapitated. The brain was extracted and fixed by immersion in 4% p-formaldehyde in 0.1 M PB. This procedure was chosen instead of perfusion, to preserve the perforant arteries and the astroglial architecture of the brain cortex. Brains were dissected and post-fixed for a week in the same fixative before cryoprotection by immersion in 30% sucrose in 0.1 M PB, pH 7.4, at 4°C, for 48 h. Brains were carved in a coronal mold with 1 mm slots (69026-Coronal RBM, Electron Microscope Sciences, Fort Washington, PA, United States) to define similar planes of sectioning between cases. The brains were then serially sectioned in the coronal plane with a sliding freezing microtome to prepare 40-μm sections (HM 430 Sliding, MICROM International, Waldorf, Germany).

#### Immunostaining

Coronal serial sections were alternately stained for Nissl with cresyl violet (C5042, Sigma-Aldrich, Merck KGaA, Darmstadt, Germany) c-Fos (226.003, Synaptic Systems, Göttingen, Germany), IBA 1 (019-19741, Wako Chemicals GmbH, Neuss, Germany) and GFAP (G6171, Sigma-Aldrich, Merck KGaA, Darmstadt, Germany). For details about antibodies used, see Table 1.

**Table 1:** Description of immunogen analysis for antibodies used in immunocytochemistry.

Antigen	Immunogen	Description	Dilution used
c-Fos	Synthetic peptide corresponding to AA 2 to 17 from rat c-Fos (UnitProt Id: P12841)	Polyclonal rabbit, Synaptic Systems Cat # 226003, **RRID:AB_2231974**	1:1000 TBS 0.05 M 1 Triton-Tx 0.3%
GFAP (glial fibrillary acidic protein)	Purified GFAP from pig spinal cord	Monoclonal mouse Sigma-Aldrich Cat # G6171, **RRID:AB_1840893**	1:500 TBS 0.05 M 1 Triton-Tx 0.3%
Iba 1	C-terminus of Iba l’ (NPTGPPAKKAISELPC’)	Polyclonal rabbit, Wako Cat # 019-19741, **RRID:AB_839504**	1:1000 TBS 0.05 M 1 Triton-Tx 0.3%

Free-floating sections were sequentially washed with 0.05 M TBS, pH 7.6, followed by endogenous peroxidase inhibition by incubation with 10% methanol + 3% H_2_O_2_ in 0.1 M PB for 10 min. Subsequently, the sections were washed in 0.1 M PB and 0.05 M TBS-Tx, pH 8.0, 0.3% Triton X-100 (T9284 Sigma, St. Louis, MO, United States) and incubated with the corresponding primary antiserum (Table 1), for 48 h at 4°C. Non-specific labeling was blocked using fetal calf serum (10%). After washing three times in TBS-Tx, for 15 min, all sections were incubated with a biotinylated secondary antibody from Vector Labs. (Burlingame, CA, United States) (biotinylated anti-rabbit IgG H+L, BA-1000 for c-Fos and IBA 1 and biotinylated anti-mouse IgG H+L, BA-2000 for GFAP) at 1:200 dilution in TBS-Tx for 120 min at room temperature. Brain sections were processed simultaneously with controls to limit confounding differences in gray level measurements caused by immunocytochemical processing. The sections were then washed with TBS-Tx and incubated for 180 min in avidin/biotin peroxidase (ABC complex, Vectastain Standard ABC kit PK-4000; Vector, Burlingame, CA, United States) and further washed with TBS-Tx, followed by Tris HCl, pH 8.0. They were then incubated in 3.3 diaminobenzidine tetrahydrochloride (DAB; D-9015; Sigma-Aldrich, St. Louis, MO, United States) with 0.006% H2O2 and 0.4% nickel ammonium sulfate to visualize the peroxidase reaction. One section per case was used as a negative control (processed without the primary antibody) to test the specificity of the immunostaining detection system.

#### Nissl Staining

The sections were stained with 1% cresyl violet (C5042, Sigma-Aldrich, Merck KGaA, Darmstadt, Germany), for 10 min. Staining was revealed in 96% alcohol + acetic acid (0.33%), and the sections were finally dehydrated in graded alcohols from 50 to 100%, followed by clearing in xylene and coverslipping.

#### Quantitative Immunocytochemistry

Dense GFAP immunoreactive products in the EES animal groups allowed us to easily delimitate the area of electric current application that we call active zone (AZ). To localize and to measure the AZ, digital image mosaics from GFAP immunoreactive serial sections were acquired at X5 using Neurolucida software (NL- Vs 8.0, MicroBrightField^®^, Inc., Williston, VT, United States) and a Leica DMRX microscope. c-Fos immunostained consecutive serial sections were acquired and also digitalized in mosaics. We chose 7 IA levels according to the Paxinos and Watson (2005) for the analysis: (1) IA 2.52–2.64 mm, (2) IA 2.88–3.00 mm, (3) IA 3.36–3.48 mm, (4) IA 4.20–4.32 mm, (5) IA 4.64–4.80 mm, (6) IA 4.92–5.04 mm, (7) IA 5.40–5.52 mm. Using Canvas software (Canvas Draw 5 for Mac), digital images were superimposed to silhouettes from equivalent IA coronal Paxinos atlas drawings, and the AC was separated from the rest of the sensory cortices in the seven coordinates of selected serial sections for analysis. Furthermore, to analyze c-Fos immunoreactivity by comparison with the ipsilateral sensory cortices, we selected a piece of tissue from the ventral limit of the secondary AC to: the dorsal mediomedial area of the secondary visual cortex in coordinate levels (1–5), to the dorsal limit of the primary somatosensory cortex in coordinate level (6) and to the dorsal limit of secondary somatosensory cortex in level (7). In addition, a digital piece of the AZ was also cut by superimposing adjacent GFAP immunoreactive sections. After this procedure, we were able to perform a uniform morphometric comparison (in group 4) between the AC, sensory cortices and AZ and between groups 2 and 4. Additionally, a copy of these digital images was used for morphometrical comparisons by layer. Based on differences in c-Fos immunoreactivity, in digital images we longitudinally cut out layers L1 to 4, L5 and L6 from sensory cortices and from the AC alone. To position the AZ on the brain silhouette, we followed the procedure previously described by Lamas et al. (2013), also detailed in a video paper (Lamas et al., 2017).

Image processing techniques were used to perform morphometric and OD analysis on c-Fos immunostained neurons. Before capturing digital images, microscope illumination was adjusted and calibrated using a stepped density filter (^®^Eo Edmund industrial opti–s – ref 32599, Karlsruhe, Germany). Photomicrographs of c-Fos immunostained sections were analyzed with ImageJ 2.0 software, using the maximum entropy thresholding segmentation algorithm and collecting values of OD, area and number of selected particles. Mean gray values of segmented neurons were converted into OD values using the ImageJ calibration plugin and standard values captured using a stepped density filter (see above). To normalize the neuronal OD values, we subtracted the mean OD of the cortex in each section from the mean OD of the segmented particle, divided by the standard deviation of OD of the entire section. The number of particles was expressed as neurons/10,000 μm^2^.

The mean and standard deviation of OD levels were assessed in the total extension of the measured sections. To cancel out differences in immunostaining intensities within and among experiments, sections with average gray levels above or below the total mean gray level plus the standard deviation were disregarded.

Details on the procedure have been previously described in the guidelines of previous papers from our group (Clarkson et al., 2010; Pernia et al., 2017).

For illustrations and topographic comparison, segmented particles prepared following the segmentation procedure explained above were saved in binary TIFF files using the ImageJ plug-in create mask.

### Statistical Analyses

Statistical analysis was performed using the IBM^®^SPSS^®^software, version 20 (IBM Corp. and SPSS Inc., Chicago, IL, United States, **RRID:SCR_002865**).

Auditory brainstem responses thresholds values before and after the surgery were compared by using a paired *t*-test analysis (groups 2, 3, and 4). Also, a paired *t*-test was used to assess wave 2 amplitudes for multisession stimulated animals, by comparing before and after EES (in groups 3 and 4).

Unpaired *t*-test was used in quantitative immunocytochemistry to analyze inter-group differences in OD, nuclei area and number of immunoreactive neurons/10,000 μm^2^ for sensory cortices and AC separately. In addition, the experimental groups were separately compared for each layer of the sensory cortices.

Furthermore, ANOVA (general linear model univariate analysis) followed by *post hoc* Bonferroni test was used for pairwise comparison of the AC, sensory cortices and AZ of the multisession-stimulated group. Differences were considered significant at *p* < 0.05.

## Results

### ABR Recordings

**FIGURE 3:**
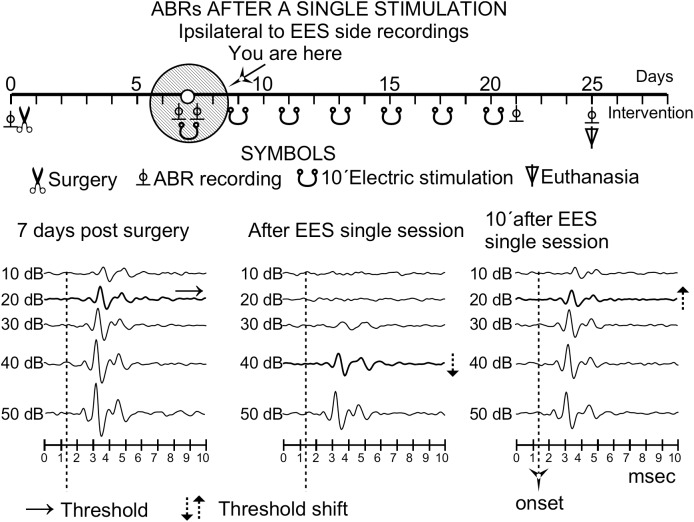
Timeline effect of a single stimulation analyzed by ABR after the first stimulus of a multisession protocol in an animal of group 3 (ipsilateral ear to EES). The time course representation is similar to that of Figure 1. Solid circles indicate the time of the recordings 7 days after surgery. Note that the 20-dB threshold shift (middle) recovers (right) 10 min after the stimulation session. Changes in threshold are indicated by doted arrows.

**FIGURE 4:**
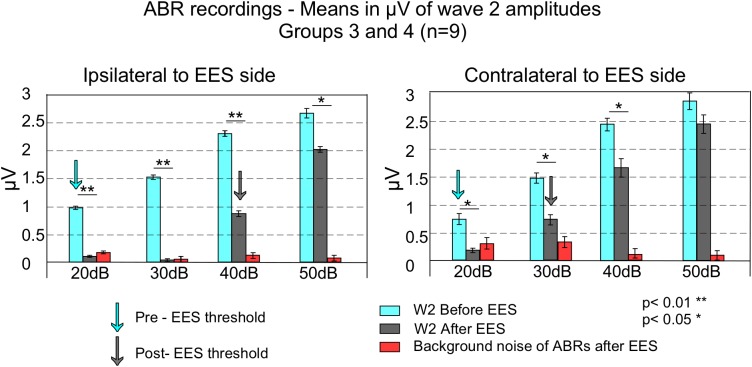
This figure shows averaged wave 2 peak to peak amplitudes and standard error of the means (error bars) of ABR of the sides ipsilateral (left) and contralateral (right) to EES, in groups 3 and 4 (*n* = 9). Blue bars represent average wave amplitude before EES and gray bars after EES. Red bars indicate background noise after EES. Before EES, ABR thresholds were established at 20 dB for both side recordings (blue arrows). Background noise to signal comparison (by comparing red and gray bars) allows to determine ABR thresholds at 40 dB in the ipsilateral side and 30 dB in the contralateral (gray arrows). EES caused stronger decreases in wave 2 amplitude in the ipsilateral side compared to the contralateral.

**FIGURE 5:**
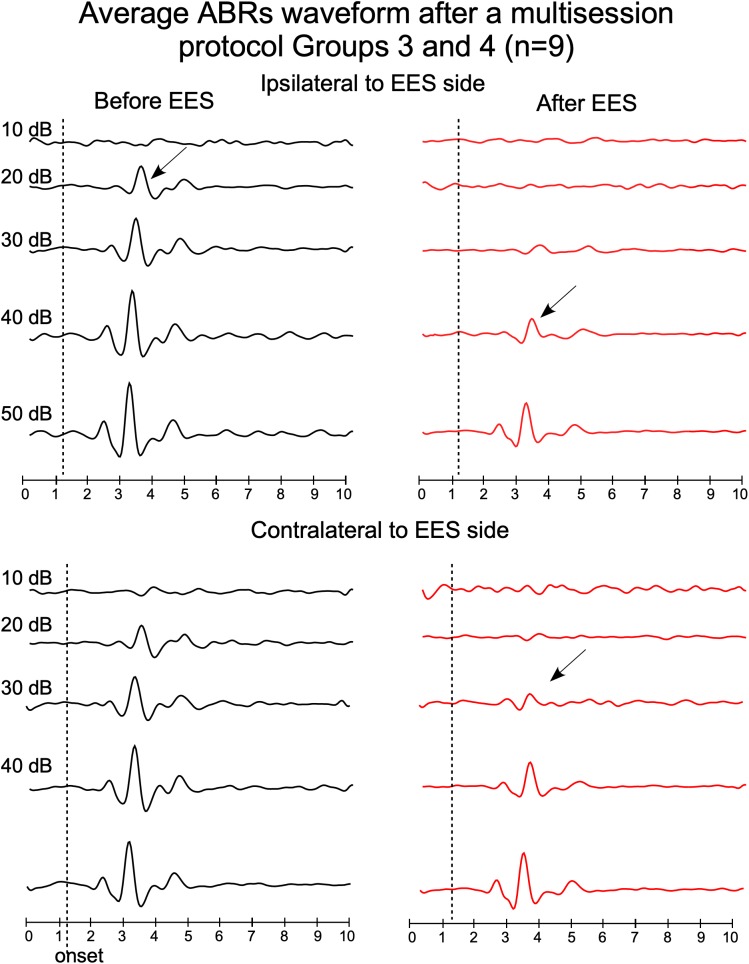
Average waveforms of ABRs from groups 3 and 4, recorded before (left) and after (right) multisession stimulation protocol (top panels: ipsilateral ABR and bottom panels: contralateral ABR, respect to the EES side). Timelines of protocol of groups 3 and 4 are shown in Figure 1. Arrows indicate average thresholds determined as indicated in Figure 4.

**FIGURE 6:**
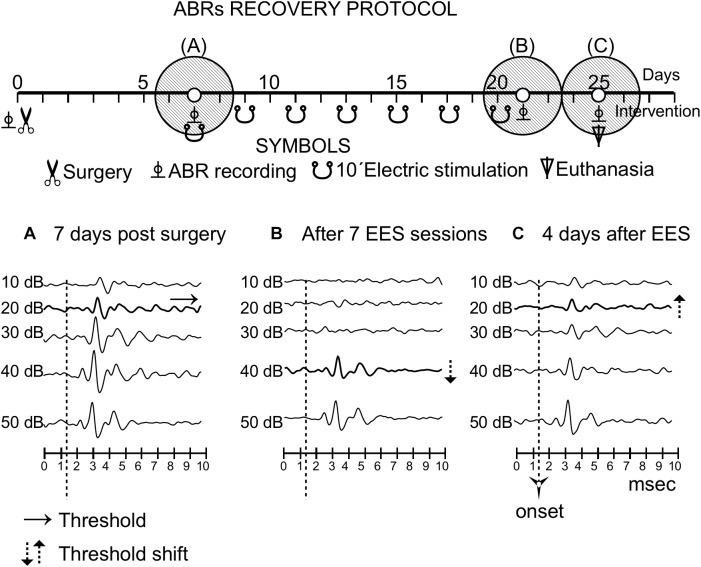
Recovery protocol of group 3. The timeline of the protocol is shown in the top of the panel, similarly to Figure 3. Three shaded circles indicate the times of the recordings, before stimulation **(A)** and (15 days) **(B)** and 4 days **(C)** after the last multisession stimulation.

Recordings made before and after the surgery showed no significant decrease in wave amplitudes or thresholds (data not shown). Control thresholds were determined to be at 10–20 dB (Figure 3–6).

After qualitative analysis of a single session stimulation in group 3 (*n* = 4), the ABR thresholds in the hemisphere ipsilateral to the EES showed an increase of approximately 20 dB; however, they recovered completely 10 min later (Figure 3).

Paired *t*-test analysis was used to compare, in animal groups 3 and 4, the thresholds before and after multisession stimulation from recordings. Wave 2 average amplitude significantly decreases in ABRs after multisession protocol in response to clicks of 20–50 dB in the ipsilateral side to EES and 20–40 dB in the contralateral (Figure 4, 5). After EES the average amplitude of wave 2 (Figure 4 gray bars) remained below the level of the 1 ms pre-stimulus background noise (red bars) for click intensities lower than 40 dB in the ipsilateral and 30 dB in the contralateral side. This asymmetry of the responses recorded at the ipsilateral or contralateral side of the EES-applied hemisphere are confirmed by wave morphology comparison as shown in Figure 5.

After the multisession protocol, 1 day after the last stimulation, ipsilateral side ABR thresholds remained 20 dB above the control levels (Figure 6). Changes in thresholds, which were analyzed qualitatively, were no longer observed 4 days later (full recovery) (Figure 6). No changes in latencies were detected in any recording (data not shown).

### Localization and Assessment of EES Effects: GFAP Immunoreactivity

**FIGURE 7:**
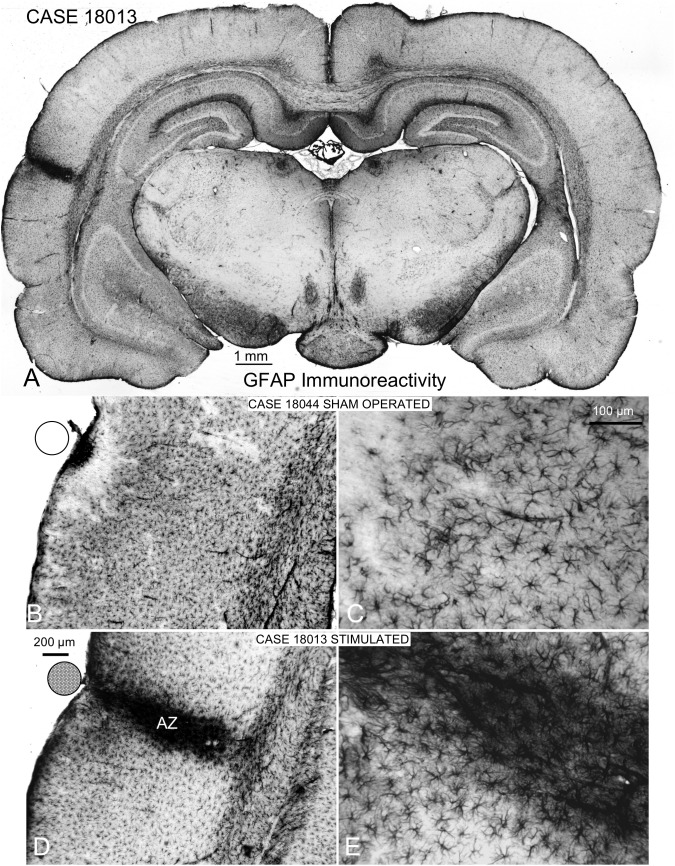
GFAP immunocytochemistry of coronal sections of multisession-stimulated and sham-operated rat brains. **(A)** Panoramic view of a case of the multisession protocol group, which shows a restricted cortical intramural increase in immunoreactivity where the stimulation electrode is located (on the AC). **(B,C)** Sham-operated control showing a small, dense immunoreactive area in the surface and a restricted, superficial band devoid of labeling. **(D,E)** Active zone (AZ) of electric stimulation. Note the intense staining of astrocytes and blood vessels inside AZ.

**FIGURE 8:**
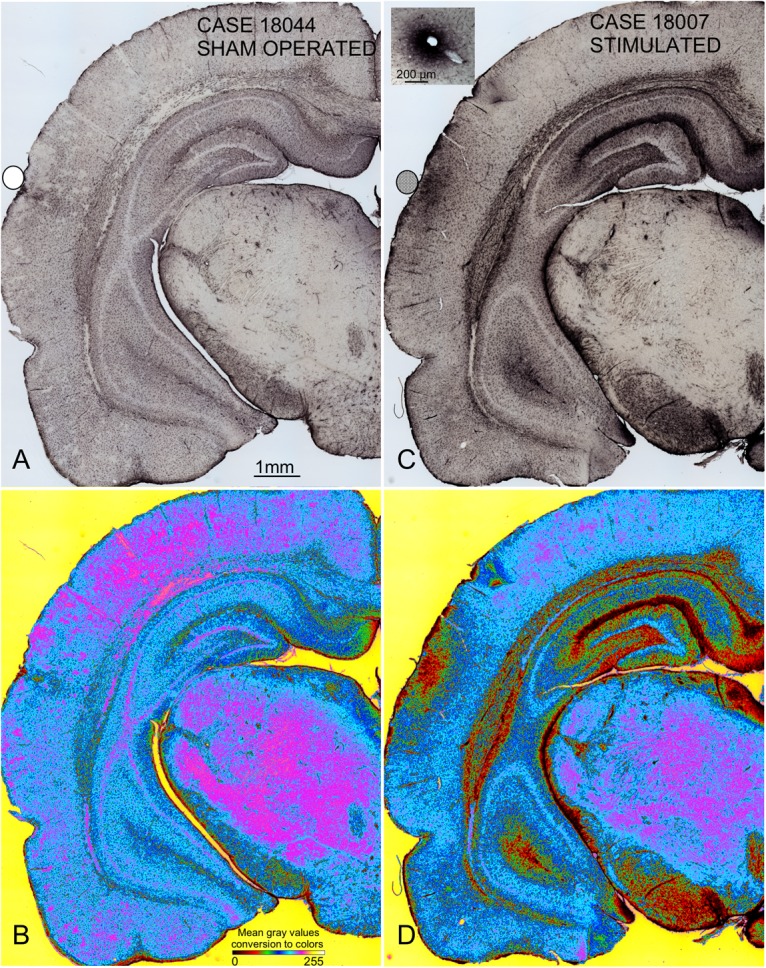
Panoramic GFAP-immunostained coronal sections from sham-operated **(A,B)** and multisession-stimulated **(C**,**D)** rat brains showing global changes in immunoreactivity only after multisession stimulation. To better visualize differences in immunoreactivity, pseudocolor-converted images (ImageJ, six shades plug-in) are shown in **(B,D)**. Codification and calibration of gray densities by color are shown in the bottom section of **(B)**. Blacks and reds match the densest immunoreactivity areas. For this analysis, digital microphotographs were taken under the same microscope illumination conditions and without subsequent manipulation. The densest immunoreactive areas found in the auditory cortex match the area of electric current injection (AZ), in the white matter and in areas of contact with the cerebrospinal fluid. The inset in **(C)** shows dense interstitial immunoreactivity around a blood vessel.

**FIGURE 9:**
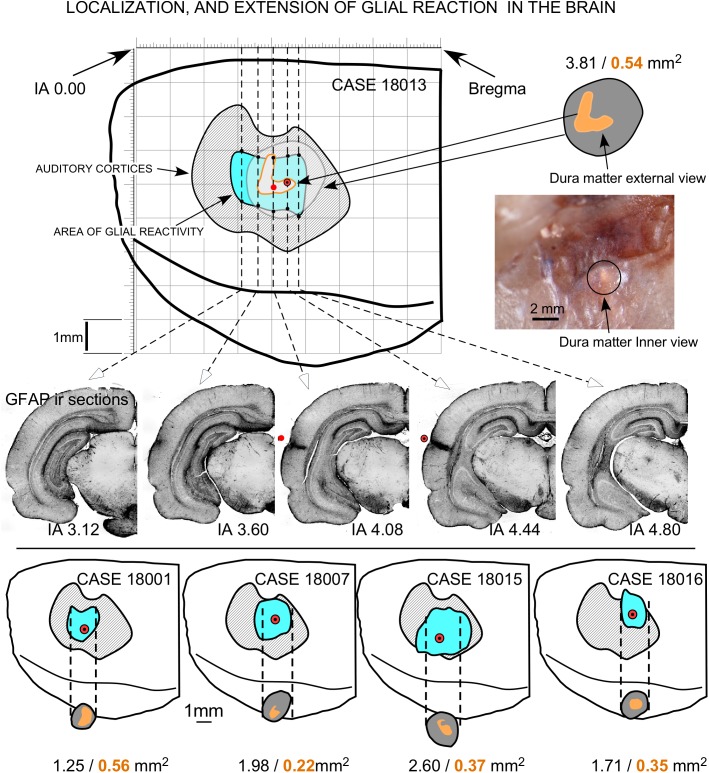
Topographical reconstruction of active GFAP immunoreactivity zones (AZ) of the brain surface, with respect to the cytoarchitectural limits of the ACs (dashed areas in gray), after multisession stimulation. The surface of the epidural area exposed to the electrode (orange areas) was estimated based on measurements taken after skull transillumination (see Figure 2). The coordinates for representation were obtained from serial sections of five coronal IA levels (middle). Drill surface and dura matter surface values are shown in black and orange letters, respectively.

Coronal sections showed a small deformation of the surface, both in sham-operated (group 2) and in multisession EES (group 4) animals, in the region of contact of the electrode with the dura (Figure 7A,B,D). The analysis of the thickness of the cortex from sham-operated rats (Figure 8A,B) showed a decrease in immunoreactivity restricted to a superficial, thin band affecting layers 1–3, with no detectable astroglial architectural reaction along cortical layers (Figure 7B,C). However, in sections of multisession-stimulated animals, a highly dense immunoreactive area of positive astrocytes and blood vessels was identified throughout the thickness of the cortex (Figure 7A,D,E). GFAP-positive glial cells and blood vessels allowed us to easily delimitate a potential area of predominance of electric field effects (AZ; Figure 7D). Astroglial cell expansions appeared orthogonally oriented, either to the surface of the brain or to perforant arteries (not shown). Extensive, dense interstitial immunoreactivity was also identified in ribbons around brain ventricles, white matter (Figure 8C,D) and around larger blood vessels (Figure 8C inset). After superimposition of coordinates taken from GFAP immunostained serial sections over a macroscopic lateral surface view of the rat brain, AZ reactive astroglial areas were always shown positioned inside the AC cytoarchitectural borders (Figure 9). Average of measurements of the surface area of the AZ (group 4) was 4.21 ± 2.06 mm^2^ (Figure 9).

### Detection of Potential EES Lesions: Nissl Staining and Iba1 Immunoreactivity

**FIGURE 10:**
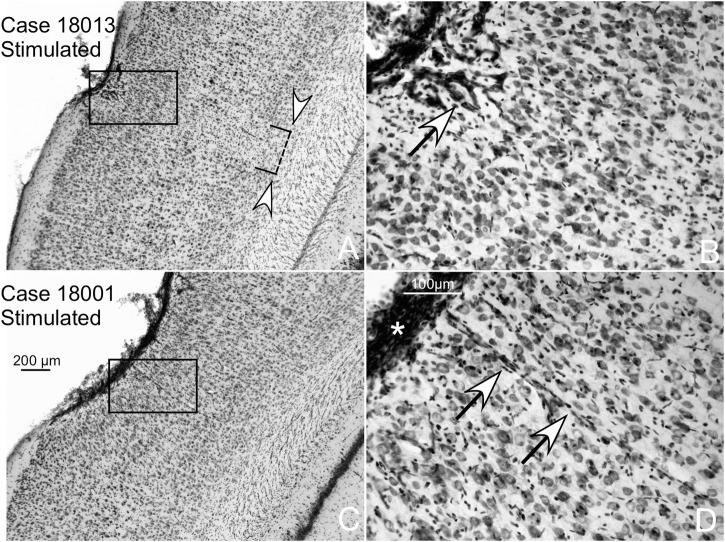
Two different cases of multisession-stimulated animals (both with more than 20 dB of increased hearing thresholds). Details of AZ in coronal, Nissl-stained sections showing small areas of neuronal loss in the surface of contact with the electrode. **(A)** White arrowheads delimitate the area of reactive gliosis and neuronal loss. **(B)** The arrow shows intense vessel staining. **(C,D)** Case with lower neuronal loss and gliosis than **(A)**. **(D)** Detail of an overstained meningeal band in relation to the contact surface of the electrode (asterisk).

In this paper, we assessed the lesion effects of electric fields by analyzing neurons in Nissl-stained sections and the potential inflammatory interstitial reaction by Iba1 immunoreactivity in microglial cells. Increased staining was shown in the area of contact of the electrode in Nissl-stained sections (Figure 10D, asterisk). Remarkably, no neuronal loss and/or increased staining or chromatolytic reactions were detected outside the AZ defined by Nissl staining and GFAP immunoreaction (please compare GFAP- and Nissl-stained adjacent serial sections from case 18013 in Figure 7, 10). Inspection of Nissl-stained sections showed an increase in the size and in nuclear staining of endothelial cells and pericytes of blood vessels within the AZ (Figure 10B,D, arrows).

**FIGURE 11:**
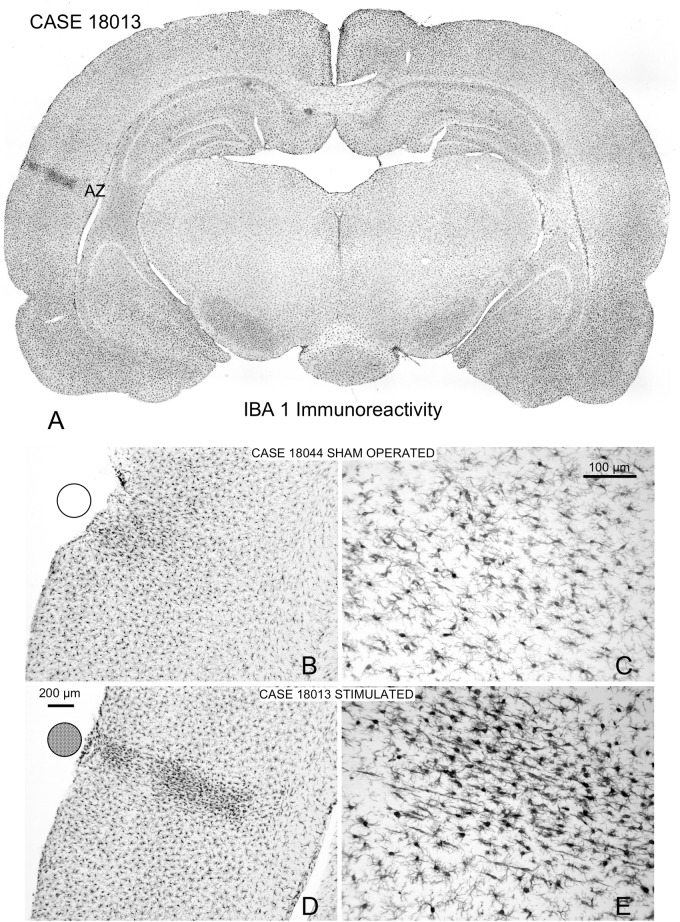
Iba1-immunostained coronal sections. **(A)** Panoramic view of a multisession stimulated animal. Microglial reactivity is shown in the contact area of the electrode (indicated by circles on the surface), which defines the AZ similarly to GFAP immunoreactivity (please compare with Figure 6). **(B,C)** Sham-operated control with a small area devoid of labeling shown in the contact area of the electrode. **(D,E)** Coronal section of a multisession-stimulated animal with positive, reactive microglial cells in the AZ distributed across layers and oriented parallel to the perforant vessel.

In Iba1 immunostained sections, microglial cells with their typical arachnoidal shape were regularly distributed along the cortex (Figure 11A). In sham-operated animals, a small group of lightly reactive cells were observed near the surface of the cortex in response to mechanical lesions (Figure 11B,C). Reactive cells can be detected in multisession-stimulated animals by the increase in the immunostaining density of the cell body and by the orientation and increased number of perisomatic expansions (Figure 11D,E). A panoramic view of reactive microglial cells allowed us to delimitate a well-defined border matching, in shape and size, areas of GFAP-reactive astrocytes of adjacent sections (AZ) (please compare Figure 11 with Figure 7).

### c-Fos Immunoreactivity

**FIGURE 12:**
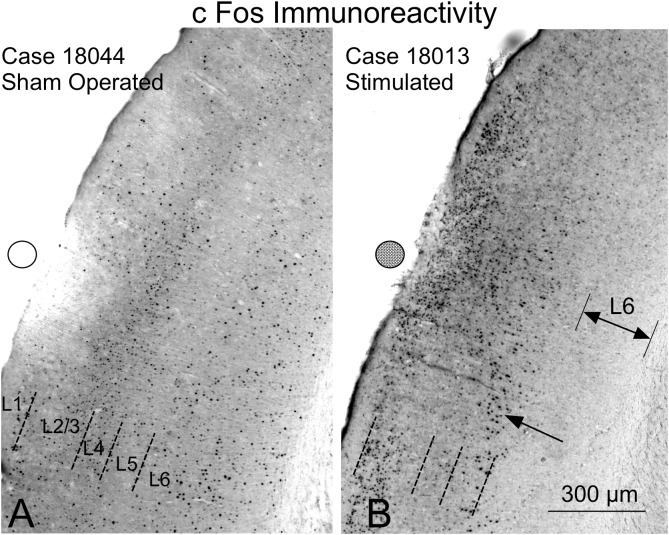
c-Fos immunoreactivity in the AC. Circles (empty – inactive, solid – active) represent the estimated position of the electrodes. The cytoarchitectural distribution of immunoreactive neurons sham-operated neurons **(A)** is different from that of multisession-stimulated neurons **(B)**. **(A)** In sham-operated controls, a small area devoid of labeling is shown in the contact area of the electrode, and positive neurons are distributed across well-defined layers. **(B)** Stimulated animals show an increase in immunoreactive neurons in the superficial area and loss of immunoreactivity in layer 6 (L6).

**FIGURE 13:**
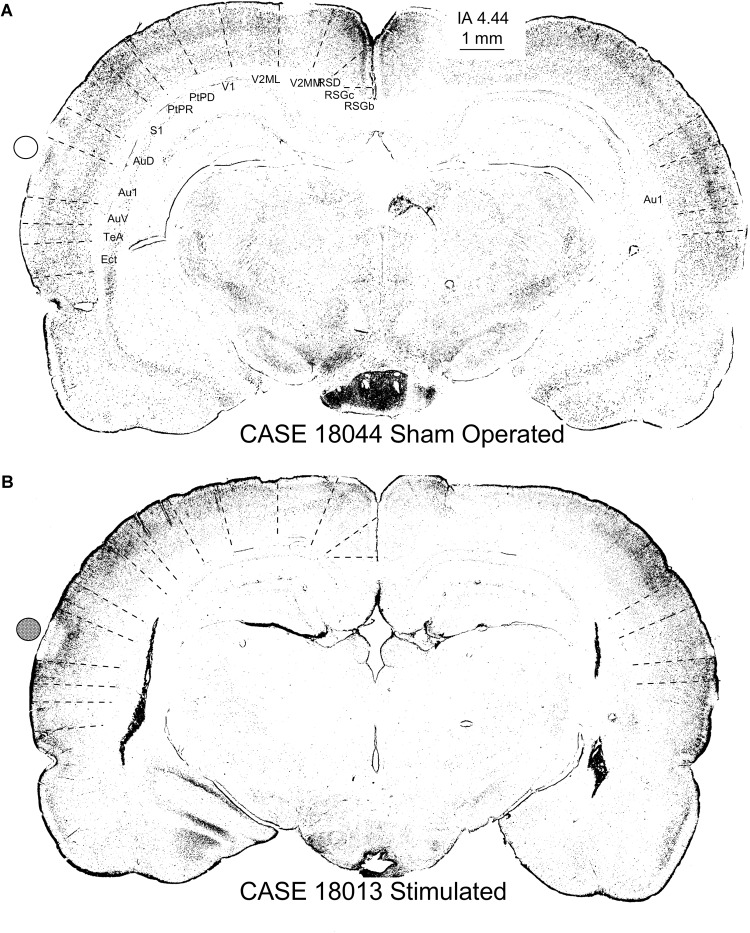
c-Fos-immunostained coronal sections of a sham-operated control **(A)** and multisession-stimulated animal **(B)** after densitometrical segmentation and map representation using the ImageJ binary mask plugin. In these figures, each dot is a c-Fos-immunopositive neuron. Sham-operated control animals in **(A)** allow us to differentiate cortical layers and the cytoarchitectural subdivision (dotted lines show superimposed and scaled Paxinos atlas cytoarchitectural subdivisions). Coronal section from a stimulated animal **(B)** shows an area devoid of labeling matching the area of contact of the electrode (active electrode position is represented by a solid circle), as well as an increase of immunopositive neurons in the supragranular layers and a decrease in layer 6. Note that cytoarchitectural subdivisions clearly delimited by differences in the distribution of c-Fos immunoreactive neurons (see the contralateral ACs in the sham control) in **(A)** are more difficult to differentiate in **(B)**. Furthermore, also note the loss of canonical organization in layers of the brain cortex, as shown in maps **(B)**, compared with the sham control **(A)**.

The nuclei of neurons that translate the c-Fos protein, turn dark after staining with 3.3 diaminobenzidine tetrahydrochloride (DAB)-peroxidase nickel (Figure 12). In the sham-operated group, in the area in contact with the electrodes, a small superficial ribbon showed a decrease in immunoreactivity (Figure 12A). Outside of this superficial region, devoid of positive neurons, immunostained nuclei were distributed along all layers of the AC (Figure 12A) as in non-operated controls (not shown). In relation to the contact of the electrode (surface deformation), in multisession-stimulated animals, layers 1–4 and particularly layer 5, showed dense immunoreactive pyramidal neurons (Figure 12B arrow), but layer 6 appeared almost unlabeled (Figure 12B – double arrow – L6). Outside the area of electrode activation (AZ), the number of immunoreactive neurons along layers decreased (Figure 12B). A remarkable change in the cytoarchitectural distribution of immunoreactive neurons along all subdivisions of the stimulated cortex was observed when inspecting the cytoarchitectural maps of segmented neurons (binary masks) (Figure 13). In these maps, each dot represents a density-gradient segmented particle and therefore the localization of an immunoreactive neuron (see “Materials and Methods” section). In sham-operated animals, well-defined layers (in particular, layer 4) were clearly distinguished (Figure 13A). In addition, differences in the distribution of immunopositive particles along the cortex allowed us to delineate cytoarchitectural subdivisions matching those defined by Paxinos (Figure 13 dotted lines). However, in multisession-stimulated animals, such cytoarchitectural organization was blurred due to the homogeneous increase in immunoreactive neurons in superficial layers of the cortex and to the loss of labeling in layer 6 (Figure 13B).

**FIGURE 14:**
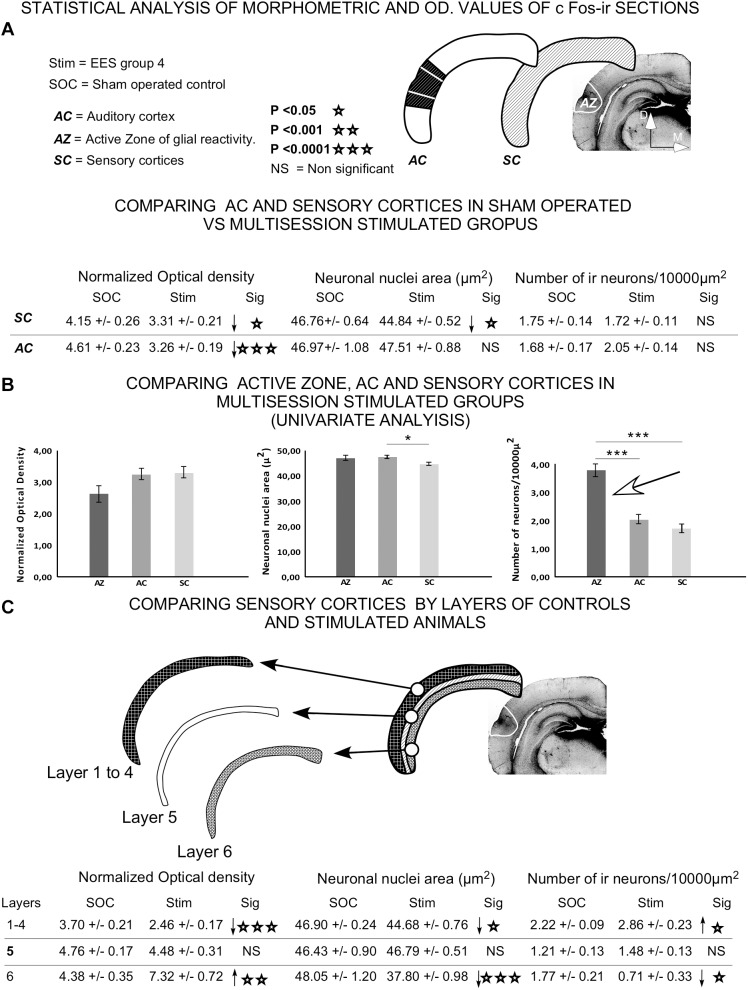
Statistical analysis of morphometrical and optical density (OD) values of c-Fos-immunoreactive neuronal nuclei. Insets represent the areas analyzed for comparison. **(A)** Statistical analysis was performed to assess significant differences in data when comparing AC or all sensory cortices (SC) between sham controls and group 4. **(B)** Univariate analysis of data between cortical areas (AZ, AC, and SC) in epidural-stimulated experimental group 4. **(C)** Differences between sham-operated group 2 and EES group 4 by layer of sensory cortices. Student *t*-test was used to compare experimental groups in **(A,C)**, and univariate general linear model was applied in **(B)**. Statistical significance was set at *p* < 0.05.

Quantitative analysis of immunoreactive neurons, after comparison of sensory cortices and AC, showed a significant decrease in OD values in multisession EES group 4 (Figure 14A) compared to sham-operated. However, the neuronal immunoreactive nuclear area decreased in the sensory cortices of stimulated animals but not in the AC (Figure 14A). No significant changes in the number of immunoreactive nuclei were observed (neither in the sensory cortices nor in the AC) when comparing sham-operated with multisession-stimulated animals (Figure 14A).

Univariate comparison of the AC, sensory cortices and AZ in multisession-stimulated animals showed a significant, two-fold increase in the normalized number of immunoreactive neurons in the AZ (Figure 14B arrow). The comparison between sham-operated and multisession-stimulated groups by layer (Figure 14C) showed no significant changes in OD, in nuclei area or in the number of immunoreactive neurons in layer 5. However, the number of neurons significantly increased in supragranular layers 1–4 and decreased in layer 6 (Figure 14C). In contrast, the OD values significantly decreased in supragranular layers 1–4 and increased in layer 6 (Figure 14C).

## Discussion

We report here that AC epidural electric activation affects short-latency brainstem responses, inducing reversible hearing suppressions (temporary threshold elevation). ABR recordings made 1 day after the end of the EES multisession protocol show a significant average increase of 20 dB in thresholds. Such threshold shift reverts to control values approximately 10 min after a single EES session and 4 days after multisession stimulation.

Multisession, direct, 0.1 mA anodal EES of the AC induces restricted astroglial, microglial and vascular reaction with no significant neuronal loss. Furthermore, the cytoarchitectural analysis of c-Fos immunoreactive neurons in the cortex after multisession EES suggest a profound and extensive change of the cortex determined by a partial loss of layering and blurring of cytoarchitectural limits of cortical subdivisions shown by c-Fos immunocytochemistry. Quantitative statistical analysis of c-Fos immunoreactivity results in a significant increase in immunoreactive neurons in the glial and vascular reactivity zones (AZ). Moreover, the OD of immunostained neurons decreased in layers 1–4, whereas the number of neurons increased. In contrast, in layer 6, the number of neurons decreased, whereas the OD values significantly increased. No changes, in any measurement parameter, were observed in layer 5.

### Auditory Brainstem Responses

The suppression and modulation effects of corticofugal pathways on cochlear responses have been shown by electrophysiological (León et al., 2012; Dragicevic et al., 2015; Terreros and Delano, 2015) molecular (Lamas et al., 2013, 2014) and behavioral (Bajo et al., 2010; Márquez-Ruiz et al., 2012) experiments in different species of mammals.

In our experiments, we have observed a very fast, 10-min recovery of thresholds after a single stimulation and recovery after comparing recordings of first and fourth day after the last stimulation in multisession animal group 3. These data suggest that EES effectively activates layer 5 neurons of the descending pathway projecting to the olivary complex (Bajo et al., 2010) and that this activation is long lasting, depending on the repetition of the stimulus. Accordingly, an increase in the density of c-Fos immunoreactive neurons of layer 5 of the AZ, which originates from the descending connections, was observed in our multisession-stimulated animals (Figure 12 arrow).

Neuronal excitability analysis after electric field stimulation has shown that direct current application in the brain causes changes in firing for several hours (Bindman et al., 1962). In addition, long-term hyperexcitability (90 min after stimulus) has been shown after motor cortex stimulation (Nitsche and Paulus, 2000). Recent studies of AMPA receptor distribution and phosphorylation after ES related to their role in NMDA-related synaptic activation support long-term potentiation induction by transcranial direct current stimulation (Stafford et al., 2017).

Despite the absence of general consensus about which nuclei or cell populations generate each wave in the ABR, it is commonly accepted that wave 2 may be generated by the activity of cochlear nuclei (presumably globular cells) after primary afferents are activated by the cochlea (Melcher and Kiang, 1996). It is known that, neurons in VNTB, which are the origin of the medial olivo-cochlear bundle (Warr and Guinan, 1979), regulate cochlear gain control by acting on the outer hair cell system in the organ of Corti (Dallos et al., 1997). Also, it is well established from classical anatomical studies, that descending projections from the AC (Feliciano et al., 1995) and also from the inferior colliculus (Vetter et al., 1993), reach the VNTB bilaterally. However, these anatomical studies also demonstrate that both descending projections (from AC and from inferior colliculus), to the VNTB have an ipsilateral predominance (see a review in Saldaña, 2015).

Wave 2 decreases in amplitude bilaterally after EES on the AC, shown here, can be interpreted as a consequence of overactivation of MOC neurons. Such effect, induced by anodal activation of the glutamatergic descending pathway, may contribute to depress the outer hair cells’ cochlear gain amplifier and, consequently, to cause a weaker activation of the cochlear nuclei which in turn induces a smaller wave 2 in the ABRs (see above). Based on the ipsilateral/contralateral asymmetry of the cortico-olivary connections mentioned above, threshold and wave amplitude differences between the ipsilateral and contralateral ABRs (see Figure 4) can be explained by a more intense activation of the neurons VNTB – MOC in the side ipsilateral to the electrically stimulated AC.

### Glial Immunoreactivity

In the CNS of mammals, increases in GFAP synthesis are detected in astroglial cells after injury or neuronal overactivation by electric stimulation (Liberman et al., 2018). Despite discrepancies among laboratories, particularly regarding results from experiments in knockout mice, GFAP is generally regarded as an intermediate filament expressed in the brain astroglial cells and extracellular matrix (Vimentin and collagen IV) (Steward et al., 1991; Eng and Ghirnikar, 1994; Galou et al., 1996; Pekny and Nilsson, 2005; Brenner, 2014; Gellner et al., 2016; Moeendarbary et al., 2017; Liberman et al., 2018). Other studies have also shown that the brain tissue increases the expression of GFAP after electric field activation (Borgens et al., 1994; Pelletier et al., 2015). In our experiments, we found an increase in the immunoreactivity in glial cells and diffusely in the interstice matching the fluid exposed surfaces of the brain (ventricles and blood vessels) after EES. Interestingly, increases in GFAP immunoreactivity and in chondroitin-sulfate proteoglycans in the neural interstitial space after a lesion have been correlated with concentration-time profiles of tetramethylammonium (Roitbak and Sykov, 1999). These experiments indicated a decrease in diffusion properties potentially related to increases in extracellular matrix molecules, which may interfere with glia-to-neuron molecular interchanges (Roitbak and Sykov, 1999). In our experiments, GFAP immunostaining after EES was correlated with electrode activation (AZ) in all elements of the NVU consisting of endothelium, glia, neurons, and pericytes. However, positive areas were also identified outside the AZ around brain ventricles.

When an electric current is applied on a liquid, positive ions drift toward the cathode, and negative ions move in the opposite direction. Their velocity (Faraday’s Laws of Electrolysis) depends on the intensity of the electric field and on the mass and charge of the ions (Semat and Katz, 1958). Such effect could be expected when an EES is applied to the cerebrospinal fluid in the brain. A similar effect can occur in the extracellular space, which accounts for approximately 12–25% of the brain volume (Peters et al., 1991). In the solid component of neural tissue, electric current propagation depends on anatomical inhomogeneities, among other factors, primarily due to myelinated fiber bundles which act as barriers to electric current propagation, as shown in slice recordings (Nelson et al., 2013). The increase in GFAP immunoreactivity reported in this paper enabled us to predict which areas of the brain have been more affected by the electric currents after EES. Accordingly, AZ draws most of the current intensity and consequently shows the densest GFAP immunoreactivity and a clear reactive response of the NVU. However, interstitial immunoreactivity around brain ventricles and blood vessels suggests a global, non-specific effect (without any NVU reactivity) most likely due to electrolytic current flows through the cerebrospinal and blood fluids.

GFAP immunocytochemistry allowed us to pinpoint that current injection affect and presumably activate the AC in our experiments. Because the neurons that form the cortico-olivary descending projection are located in the deeper infragranular layers 5 and 6 (Beyerl, 1978; Games and Winer, 1988; Weedman and Ryugo, 1996), the changes in threshold in ABRs we have demonstrated the effectiveness of EES across all layers of the cortex. The ABR changes correlated with the AZ, as defined by GFAP immunoreactivity, suggest that the overstained NVU can be related with the EES-induced increase in neuronal activation (Steward et al., 1991; Dallérac et al., 2013). However, AZ-positive, GFAP overstaining of the NVU can also be explained by a tissue reaction to eliminate debris to the Virchow Robin compartments after small mechanical or electrical lesions (Marín-Padilla, 2012). Although mechanical or electrical lesions cannot be ruled out, our Iba1 (slight reactive profiles of microglial cells) and Nissl staining (no appreciable chromatolytic figures) results support neuronal overactivation as the main promoter of glial reactivity in the AZ.

The GFAP and Iba1 immunocytochemistry analysis reported in this paper highlights an intracortical transmural effect, along the thickness of the cortex, as well as an electrolytic effect on the brain surface. Although the former induces a restricted, intense, local glial reaction, the latter effect affects the surface of the brain more softly, albeit extensively.

### c-Fos Immunoreactivity

Changes in c-Fos immunoreactivity have been evaluated as a neuronal activity and plasticity marker, because c-Fos is overexpressed upon neuronal depolarization and firing, synaptic stimulation and growth factor upregulation (Sheng and Greenberg, 1990; Joo et al., 2015). Notwithstanding c-Fos expression is also associated with neuronal injury (Bullitt, 1990; Chen et al., 2015) and apoptosis (Preston et al., 1996). Furthermore, we have demonstrated that c-Fos is a useful tool to analyze cytoarchitectural changes in the cortex when showing specific global increases in the number of neurons in the visual cortex after cross modal invasion, in a model of long-term bilateral deafness (Pernia et al., 2017).

Although chromatolytic lesions were scarce in our Nissl material (see above) and the number of neurons in the cortex does not significantly change after EES, our changes in c-Fos labeling primarily result from neuronal synaptic plasticity and activity regulation. Accordingly, growth factors such as BDNF, whose expression has been correlated with c-Fos (Dong et al., 2006), have been postulated as key mediators of synaptic plasticity after anodal current stimulation (Fritsch et al., 2010). Local increases in c-Fos immunoreactivity in AZ neurons could be associated with a direct effect of anodal currents on neuronal activation. However, outside the AZ, a predominantly unexpected redistribution of c-Fos immunoreactive neurons in supragranular layers, which we interpreted as a consequence of electrolytic activation, has also been shown along the cortex. Nevertheless, the activation of neighboring cortices by the system of horizontal cortical connections as an explanation for c-Fos cytoarchitectural changes after current stimulation could not be ruled out. Paired restricted injections of fluorogold and diamidine yellow in somatosensory, auditory and visual cortices have demonstrated an extensive system of horizontal connections between sensory cortices in the rat (Paperna and Malach, 1991). Moreover, in rats guided by intrinsic signal optic imaging, small pressure injections of biotinylated dextran amino or cholera toxin delivered in layers 1–3 of sensory cortices have demonstrated long-distance horizontal connections and axonal lengths up to 2 mm (Stehberg et al., 2014).

We suggest here that both electrolytic activation and intrinsic horizontal cortical interconnections may contribute to the cortical cytoarchitectural changes we have shown by c-Fos immunocytochemistry. Nevertheless, further studies are required to confirm these findings.

Interestingly, the comparison of quantitative data by layer indicated that layers 1–4 showed a significant increase in immunoreactive neurons and that layer 6 showed a significant decrease, with no significant changes in layer 5. As initially shown by Cajal and most recently by other authors (Mitani et al., 1985), apical dendrites of infragranular pyramidal neurons cross the entire cortex and extensively ramify in more superficial layers, particularly in layers 1 and 2. The neurons of layer 1 are relatively sparse, with an estimated density of 1,173 neurons/mm^3^ (Gabbott and Somogyi, 1986) and GABAergic neurons account for 90% of this neuronal population (Prieto et al., 1994). Recent studies of 3D reconstructions demonstrate that GABAergic neurons in layer 1 are highly connected by multiple synapses with an average of nine putative synapses per connection (Muralidhar et al., 2014). Functional recordings after delivering the GABA blocker picrotoxin shows larger field potential in deeper layers II/III, thus demonstrating the inhibitory descending intracortical effect of layer 1 networks (Shlosberg et al., 2003). In addition, the extensive inhibitory network of layer 1 is interconnected with and coupled by an extensive system of electrical low resistance unions (gap junction – Efapsis). Furthermore, a recently published study has shown that a reduction in this electrical coupling, which can be induced by our electrical stimulation, reverses in an increase in excitatory synaptic inputs to layer 1 interneurons (Yao et al., 2016). As a working hypothesis, we speculate that electrolytic activation of superficial layers after EES, shown in our GFAP material, may induce overactivation of layer 1 networks, thereby inducing inhibition on the apical dendrites of layer 6 pyramidal neurons. Paired cross-correlation of single unit recordings in layers 1 and 6 after EES should be performed to analyze in depth this problem.

## Conclusion

In conclusion anodal direct current (0.1 mA) epidural multisession stimulation of the AC reversibly decreases hearing sensitivity. Furthermore, our results indicate that such repetitive stimulation induces a local activation of AC as well as an extensive superficial electrolytic activation of the brain cortex.

Future clinical applications of either invasive or non-invasive electrical stimulation must consider the safety of patients subjected to global and non-specific electrolytic activation of the brain. Finally, a reversible decrease in hearing sensitivity to sound after EES may motivate otologists to look for clinical applications in hyperacusis, tinnitus or otoprotection.

## Ethics Statement

This study was conducted according to Spanish (Royal Decree 53/2013 – Law 32/2007) and European Union (Directive 2010/63/EU) guidelines for the care and use of laboratory animals. Protocols were approved by the Ethics Committee on Animal Experimentation of the University of Salamanca (Permit Number: 2012–265).

## Author Contributions

MM, JD-G, and MP designed the experiments. AC-R, ID, IP, MP, and MM performed the experiments and analyzed the data. IP contributed by performing histological methods. ID conducted the quantitative immunocytochemical study. ID and JC performed the statistical analysis. AC-R and DP-G contributed to the analysis of ABRs. All authors participated in the discussion of the experiments. MM wrote the manuscript.

## Conflict of Interest Statement

The authors declare that the research was conducted in the absence of any commercial or financial relationships that could be construed as a potential conflict of interest.

## Abbreviations

ABRs: auditory brainstem responses
AC: auditory cortex
AZ: active zone
BDNF: brain-derived neurotrophic factor
CAP: compound action potential
CM: cochlear microphonic
CNS: central nervous system
DPOAE: distortion-product otoacoustic emissions
EES: electrical epidural stimulation
GFAP: glial fibrillary acidic protein
IA: interaural
MOC: medial olivo-cochlear nucleus
NVU: neurovascular unit
OD: optical density
PB: phosphate buffer
SC: sensory cortices
TBS: Tris-buffered saline
TBS-Tx: Tris-buffered saline + 0.3% Triton X-100
TTX: tetrodotoxin
VNTB: ventral nucleus of the trapezoid body.
